# Prevention and Control of Losses of Gaseous Nitrogen Compounds in Livestock Operations: A Review

**DOI:** 10.1100/tsw.2001.339

**Published:** 2001-11-27

**Authors:** A.A. Jongebreur, G.J. Monteny

**Affiliations:** Institute of Agricultural and Environmental Enginering (IMAG B.V.), Wageningen University and Research Centre, Wageningen., The Netherlands

**Keywords:** ammonia emission, greenhouse gas emissions, odour emissions, climate change, animal housing systems, manure storage, manure treatment, volatilisation of ammonia, sustainable livestock production

## Abstract

Nitrogen (N) losses from livestock houses and manure storage facilities contribute greatly to the total loss of N from livestock farms. Volatilisation of ammonia (NH_3_) is the major process responsible for the loss of N in husbandry systems with slurry (where average dry matter content varies between 3 and 13%). Concerning this volatilisation of NH_3_, the process parameters of pH and air temperature are crucial. During a period of approximately 10 years, systematic measurements of NH_3_ losses originating from a large variety of different livestock houses were made. One of the problems with NH_3_ emissions is the large variation in the measured data due to the season, the production of the animals, the manure treatment, type of livestock house, and the manure storage. Generally speaking, prevention and control of NH_3_ emission can be done by control of N content in the manure, moisture content, pH, and temperature[1]. In houses for growing pigs, a combination of simple housing measures can be taken to greatly reduce NH_3_3 emissions[2]. In houses for laying hens, the control of the manure drying process determines the emission of NH_3_[1]. Monteny[3] has built an NH_3_ production model with separate modules for the emission of the manure storage under the dairy house and the floor in the house. Manure spreading is also a major source of NH_3_ emission and is dependent on slurry composition, environmental conditions, and farm management. The effects of these factors have been employed in a model[4]. Losses via NO, N_2_O, and N_2_ are important in husbandry systems with solid manure and straw. The number of experimental data is, however, very limited. As N_2_O is an intermediate product of complex biochemical processes of nitrification and denitrification, optimal conditions are the key issues in N_2_O reduction strategies. We may expect that in the near future the emission of greenhouse gases will get the same attention from policy makers as NH_3_. Sustainable livestock production has to combine low emissions of gaseous N compounds with acceptable odour emissions, low emissions of greenhouse gases, and acceptable standards of animal welfare. For the entrepreneur, the strategy must be built on the regulations, the special conditions of his farm, and what is reasonably achievable.

## INTRODUCTION

During the past decades, livestock production has been intensified, both in numbers of livestock and in production level, with an increased input of minerals through feedstuffs and chemical fertilisers. As a consequence, emissions of ammonia ($NH_3$), odours, and greenhouse gases to the atmosphere from livestock production sources (housing systems, manure storage, land spreading of manure, grazing) have increased drastically. The emission of $NH_3$ from agricultural activities in Europe, excluding the former USSR, doubled between 1950 and 1986[5], whereas, for The Netherlands, this increase was by a factor of 2.5[6]. This increased $NH_3$ emission has substantially contributed to the exceeding of critical loads for nitrogen (N) deposition in many European countries, leading to eutrophication and soil-acidification related environmental stress[7,8]. In The Netherlands, for example, about 46% of the potential acid deposition is caused by emission of $NH_3$[9], mainly originating from agriculture. The $NH_3$ emission from agricultural sources was estimated to be 180 kton in 1995[10]. According to the National Environmental Policy Plan[11], the Dutch governmental policy aims at a reduction, in the year 2010, of 70% and, for the year 2030, of 75 to 85%.

Since 1972, the odour nuisance from agricultural sources has generally been controlled by a regulatory system based on distance zones and odour strength. In the 1980s, results of odour measurements, dispersion modelling, and neighbourhood enquiries have provided a more quantitative basis to this guideline.

Methane ($CH_4$) and nitrous oxide ($N_2O$) contribute to global warming. The global warming potential (GWP) of $CH_4$ and $N_2O$ is estimated to be 20[12] and 300 times the GWP of carbon dioxide ($CO_2$). Furthermore, $N_2O$ emissions contribute to depletion of ozone in the stratosphere, via stratospheric conversion of $N_2O$ to NO[13].

A current estimate of the global emissions of $N_2O$ is 17.7 MT (1 MT = Tg = $10^{12}$ g), with 8.0 MT per year being emitted from anthropogenic sources, of which 6.2 MT is from livestock production[14]. Olivier et al.[13] indicated that fertiliser consumption and animal excreta are equally important and are the largest contributors to agricultural $N_2O$ emissions. Many authors mention the great uncertainty in the greenhouse gas emission data[12,15,16], mainly caused by lacking information about emission factors for the various sources. In The Netherlands the emissions of greenhouse gases from agricultural sources in 1999 were 25.8 billion $CO_2$ equivalents, or approximately 11% of the total greenhouse gas emissions. The State Institute for Public Health and Environment in The Netherlands (RIVM)[10] is expecting a decrease of 10 to 15% in the greenhouse gases from agriculture.

In this paper different types of emissions, mainly N, are put in the context of sustainable agriculture and food production.

## PROCESSES AND PARAMETERS INFLUENCING EMISSIONS FROM AGRICULTURAL SOURCES

Urea hydrolysis, catalysed by the enzyme urease, follows the Michaels Menten kinetics for basic enzymatic conversion processes[17]. Urease is produced by microorganisms that are abundantly present in faeces and, thus, also upon surfaces that are frequently fouled with faeces, like floors[18]. Eq. 1 represents urea hydrolysis in a liquid environment (e.g., urine on the floor, urine in the straw bed, or slurry in the pit).

$$CO(NH_2)_2 + H_2O \xrightarrow{\text{urease}} 2NH_3 + CO_2 \qquad (1)$$

The rate of urea hydrolysis and, thus, the ammonia ($NH_4^+$) production rate depend on the urea concentration in the urine and the maximal rate of enzymatic urea hydrolysis at high urea concentrations, also called “urease activity”.

In the liquid, ionised $NH_4^+$ and unionised $NH_3$ are in equilibrium (Eq. 2).

$$NH_3 + H_2O \xleftrightarrow{\text{pH,T}} NH_4^+ + OH^- \qquad (2)$$

The amount of $NH_3$ relative to total ammoniacal N (TAN: sum of $NH_3$ and $NH_4^+$) in the liquid is determined by the acid dissociation constant ($K_a$) for $NH_3$ and by the pH[19].

Volatilisation of $NH_3$ is by convective mass transfer, from the boundary of urine or slurry and air, to the air above the floor or above the slurry in the pit. The amount of volatile $NH_3$ depends on equilibrium between $NH_3$ in the liquid (l) and in the gas phase (g) at that boundary (Eq. 3), following Henry’s Law.

$$NH_3(l) \xleftrightarrow{\text{T}} NH_3(g) \qquad (3)$$

This equilibrium is temperature-dependent; higher temperatures result in a higher amount of gaseous $NH_3$.

$NH_3$ volatilisation rate (Eq. 4) is the product of the $NH_3$ mass transfer coefficient and the difference in concentration or partial

**TABLE 1**
**Fraction of TAN Present as $NH_3$ as a Function of pH and Temperature, with TAN as the Sum of $NH_3$-N and $NH_4^+$-N in Liquid[3]**

| | Temperature (°C) | | | | |
|---|---|---|---|---|---|
| pH (-) | 0 | 5 | 10 | 15 | 20 |
| 7.5 | 0.07 | 0.1 | 0.13 | 0.18 | 0.26 |
| 8.0 | 0.21 | 0.29 | 0.41 | 0.57 | 0.80 |
| 8.5 | 0.66 | 0.92 | 1.3 | 1.8 | 2.5 |
| 9.0 | 2.1 | 2.9 | 4.0 | 5.5 | 7.5 |
| 9.5 | 6.2 | 8.5 | 11.0 | 15.4 | 20.0 |

pressure of gaseous $NH_3$ (g) between boundary (bound) and air above the boundary (air).

$$NH_3(g,bound) \xleftrightarrow{T,v} NH_3(g,air) \quad (4)$$

The mass transfer coefficient for $NH_3$ depends on temperature (T) and air velocity (v) at the boundary.

Other than $NH_4$, $N_2O$ is not directly produced from excretia. Details of the processes and the process conditions of $N_2O$ production are, in general, poorly understood[20,21] and may be more complex than for $NH_3$. Before $N_2O$ is emitted from slurry/manure, ammonification of urea (either directly from urine from ruminants, or indirectly, through conversion of uric acid to urea, in excreta from birds) has to take place first. The ammonification process is well understood and described for urine[22] and uric acid[23], which are excreted by cattle/pigs and poultry, respectively. The $NH_4^+$ that is produced is transformed by nitrifying bacteria in the presence of a sufficient supply of oxygen (nitrification; Eq. 5).

$$NH_4^+ \xrightarrow{\text{Nitrosomon asspp.}} NO_2^- \xrightarrow{\text{Nitrobacterspp}} NO_3^- \quad (5)$$

$N_2O$ is not an intermediate product of nitrification under optimal conditions. It may be produced only under conditions of low oxygen availability, as a consequence of reduction of oxidised N compounds ($NH_2OH$, $NO_2^-$)[24]. Furthermore, high $NH_3$ concentrations and low C to N ratios negatively affect the biochemical transformation of $NH_4^+$ to nitrite/nitrate and, thus, the production of $N_2O$.

The denitrification process (Eq. 6) takes place in treated slurry (e.g., nitrification of $NH_4^+$ by oxidation/aeration, or in soils where nitrate from chemical fertilisers is potentially transformed to N gas, with $N_2O$ as one of the intermediate products).

$$NO_3^- \rightarrow NO_2^- \rightarrow NO \rightarrow N_2O \rightarrow N_2 \quad (6)$$

Concerning $N_2O$ production in soils, and presumably also for other on-farm subsystems, critical factors for denitrification are the presence (or lack) of denitrifiers, which represent a large spectrum of heterotrophic bacteria, oxygen, nitrite, and nitrate, and easily oxidisable organic matter (C-source for bacteria[21]).

## TECHNOLOGY AND TECHNIQUES TO REDUCE AND PREVENT $NH_3$ EMISSIONS

The amount of $NH_3$ emitted to the atmosphere on a global scale is estimated at 54 million tons of N per year (range: 23 to 88), of which 22 million tons (range: 20 to 61) originates from animal husbandry[13,33]. In Europe and The Netherlands, 53 and 60%, respectively, of the agricultural $NH_3$ emissions originate from cattle husbandry[34,35]. Pig and poultry husbandry are responsible for the remainder. Although great variation exists, the relative contributions of the livestock house with the outside storage, slurry application, and grazing to the total $NH_3$ emission from animal husbandry are approximately calculated at 50, 40, and 10%, respectively, for situations where no emission-reduction measures are taken[36,37].

The Dutch $NH_3$ emission reduction goal is 70% in the year 2010. This means that $NH_3$ emissions from all sources, e.g., land spreading slurry, animal houses, slurry storages, grazing, must be substantially reduced.

### Effect of Dairy Cow Housing Systems

$NH_3$ emission from cattle housing systems, including storage, is estimated (not measured!) to be 28% of the national emission[35]. In addition to the legally required advanced slurry application techniques and covering of outside storages, legislation is being prepared for animal houses. Besides the measurements of $NH_3$ emission through air exchange rate and $NH_3$ concentration, $NH_3$ emissions at the farm level can be estimated by calculation of the N flows. The emission fraction represents the amount of $NH_3$ emitted from each component of the N cycle and is defined as the percentage of the N excreted in the cow house or during grazing, in the slurry storage, and during and after land application. In The Netherlands, the emission fraction for cows in cubicle houses is normatively set at 10.2%, whereas the emission fraction is 7.1% for tie stalls[3,37,38]. Reduction of $NH_3$ emission can be achieved by measures based on engineering, nutrition, and management. The main principles are reduction of the urea concentration of urine by nutritional measures, dilution of urine on floors and removal from floors, slowing down the urea hydrolysis on floors, control of pH, reduction of mass transfer of $NH_3$ from urine and slurry, and reduction of air exchange between house and pit. In Table 2, these methods and their measured maximal reduction are summarised.

It may be concluded that the floor system and, related to this, the removal of the slurry from the house are main factors in $NH_3$ emission rates. $NH_3$ emission from tie stalls (5 to 27 g per day per cow)[40,41,42,43] is lower than from loose housing systems (20 to 45 g per day per cow)[40,44]. Urea concentration in the urine, urease activity, pH, temperature, air velocity, and area of emitting surfaces (floor, pit) are parameters influencing emission of $NH_3$. The slurry pit contributes, on average, 25 to 40% to the $NH_3$ emission from cubicle dairy cow houses with slatted floors, with a maximum percentage of 80% in situations with great differences in temperature between outside air and air in the slurry pit. The potential of feeding management by changing the diet of the dairy cow is high[3,45]. The so-called Green Label Awards were introduced during the last part of the past century to stimulate investments in these housing systems on a voluntary basis. Compared to traditional housing systems, the Green Label houses have to reduce $NH_3$ emission by at least 50%, without causing a shift to other sources of environmental pollution. To stimulate sustainable agriculture in topics other than the environment, e.g., animal welfare, food safety, labour condition, this Green Label system will be transformed. In practice it can be seen that only a few systems comply with the reduction percentage of $NH_3$ emission of 50%. Only cubicle houses with an adapted floor design, e.g., grooved floors, are being built in larger numbers[46].

### Effect of Slurry Application Method

Surface spreading of slurry inevitably leads to emission of $NH_3$ into the air. Injection of slurry on grassland reduces the emission

**TABLE 2**
**Overview of the Working Principle of Emission-Reducing Measures and Reduction of the $NH_3$ Emission from Dairy Cow Buildings Reported in Literature (in % Compared to Slatted Floors)**

| Measure | Process involved | Control factor | Maximal reduction | Reference |
|---|---|---|---|---|
| **Feeding strategies** | urine and faeces | urea concentration | 39 | 25 |
| **Slurry Handling:** | | | | |
| * flushing with water | enzymatic conversion | urea concentration | 17 | 26 |
| * formaldehyde flushing | enzymatic conversion | urease activity | 50 | 26 |
| * slurry acidification | dissociation | pH | 37 | 27 |
| + additional flushing | dissociation | pH | 60 | 28 |
| **Housing and floor systems:** | | | | |
| * V-shaped solid floors | air exchange/ volatilisation | air velocity | 52 | 29 |
| + flushing with water | enzymatic conversion | urea concentration | 65 | 30 |
| + formaldehyde | enzymatic conversion | urease activity | 80 | 27 |
| *reduced slatted floor area | volatilisation | emitting area of floor/pit | 10 | 31,32 |
| *tie stalls | volatilisation | emitting area of floor/pit | 28 | 31 |

of $NH_3$. However, application of deep-working injector tines requires high draught forces. New techniques are available to prevent the mentioned problems, e.g., shallow injection, with open slot and closed slot, and narrow band spreading. With the help of these techniques, reductions in $NH_3$ emission of 70 to 95%[4] can be achieved, in comparison with surface spreading. Different factors influence the reduction percentage, e.g., slurry composition, weather conditions, soil type, and farm management. Researchers have modelled the effects of these different parameters for each application technique. Some of these factors seem to have a major effect on the $NH_3$ emission. The mentioned new techniques show a more uniform distribution pattern of the slurry than broadcast surface spreading[4].

## Effects of Pig Housing Systems, Nutritional Factors and Management

$NH_3$ volatilisation in pig houses has to be prevented to achieve air quality improvement and the protection of the environment, due to the acid deposition. $NH_3$ mainly volatilises from urine puddles on the floor and from the slurry pit under the floor. As described above, the volatilisation process is influenced by several factors. The main strategies to reduce $NH_3$ emission by dietary composition are shifting N excretion from urine to faeces, reducing the pH of the slurry in the pit, and decrease of the intake of dietary N[47]. By including nonstarch polysaccharides, the $NH_3$ concentration is lowered and the pH of the slurry is decreased through the formation of fatty acids. The $NH_3$ emission from houses for rearing and fattening pigs can be decreased to almost 50% through reduction of the slatted floor area to 25%, in comparison with the slatted floor area of 50%. Fouling of the pen area must then be prevented by an optimal pen design and integrated with ventilation with low inlet and low outlet. The combination of housing and nutritional measures also shows promise[2].

$NH_3$ concentrations show large variation, mainly caused by design and lay-out of the building, ventilation method, farm management, N intake via the ration, and the climatic conditions inside and outside of the facilities[40]. Straw systems have not been investigated much in The Netherlands. Measurements of deep litter systems for rearing and fattening pigs showed that the $NH_3$ emission was only slightly reduced in comparison with fully slatted floors, whereas an increase of the emission of greenhouse gases occured. One approach for the design of sow facilities is the reduction of the emitting area[49]. The design for group housing of sows, with a complete functional separation of the lying area with straw and the activity area with concrete slats and solid floor, is currently being evaluated[50]. The application of flushing systems, where frequent removal of slurry is combined with dilution, also reduces the $NH_3$ emission from the pig house[51]. Recent research relating $NH_3$ and odour emission from pig housing systems has shown that measures taken to reduce $NH_3$ emission also decrease the odour emission substantially[52]. End-of-pipe techniques, such as biological scrubbers and biological filters, are known for their high emission-reduction potential[53].

## Effects of Housing Systems and Manure Treatment for Poultry

To achieve low $NH_3$ emissions from poultry houses, the control of the dry matter content of the litter is crucial. This is possible using modern techniques. In aviary systems, regular removal of manure and drying of the litter can achieve low emission comparable to the cage system, with improved drying of manure on the belt[54]. Improvement of animal welfare conditions can thus be combined with low $NH_3$ emissions. For broilers, a system has been designed with litter drying, but not applied due to costs, hygiene concerns, and energy consumption. End-of-pipe techniques, e.g., bio-scrubbers and biofilters, have a high potential for the reduction of $NH_3$, dust, and odour. However, the low-$NH_3$-emitting poultry systems do not show substantial reductions in odour emission, compared with traditional systems[52]. In the poultry industry, methods for further drying of the manure and burning and gasifying of the dry manure in energy producing plants are being developed.

In 1999 the E.U. approved a Directive[55], which bans the use of cages after 2012, and allows no new investments in cages after 2003.

## SOURCES OF $N_2O$ IN ANIMAL HUSBANDRY

### Animals and Animal Houses

Although $N_2O$ may be emitted from animals through either breathing or flatus, Kroeze[56] indicated that the contribution of $N_2O$ from the animals' digestive systems is not known and may be negligible on a national scale. $N_2O$ emissions from animal houses are therefore likely to originate from the animal excreta stored indoors.

$N_2O$ is not likely to be produced from slurry stored indoor in pits beneath the slatted floors. (This is the most common excreta storage system in pig and cattle husbandry.) However, emissions of $N_2O$ (and $CH_4$) are to be expected in housing systems that are based on solid manure (FYM = Farm Yard Manure).

In pig husbandry, a combination of litter-based housing with microorganisms added to enhance conversion of $NH_3$ to microbial protein was developed and investigated several years ago[48]. Measurements showed that 15 to 21% of the slurry N may be emitted as $N_2O$[48,57]. These and other results from $N_2O$ emission studies in pig houses are summarised in Table 3.

The $N_2O$ emissions from housing systems for fattening pigs without straw are, on average, around 0.2 kg per place and year for housing systems, whereas values range between 0.6 and 3.7 kg per place and year for deep litter systems and systems with straw. For poultry housing systems with straw, $N_2O$ emissions ranging from 0.02 to 0.15 kg per animal place per year were reported by Mennicken[60].

### Outdoor Slurry Storage

$N_2O$ production from outdoor manure and slurry storage is similar to indoor storage. Thus, $N_2O$ emissions are to be expected only from stored solid manures (e.g., FYM). The most important differences between indoor and outdoor storage are the method of storage and the storage conditions. Most indoor storages (slurry pits) are relatively open to the air inside the animal house, and gases produced are likely to volatilise to the air above the stored excreta. In several European countries, however, some sort of covering for outdoor slurry storage is advised (e.g., in Denmark, U.K.) or enforced by law (e.g., in The Netherlands) to reduce the emission of gases to the atmosphere.

Sibbesen and Lind[24] reported a value of 0.3 g of $N_2O$ per day per $m^2$ of FYM (from pig slurry) stored under summer conditions, whereas Sommer et al.[61] found $N_2O$ emission rates of 0.73 g per day per $m^3$ of cattle slurry during summer storage. Both sets of data indicate that $N_2O$ emissions from storage of solid manure are not to be neglected and need further attention.

### Slurry/Manure Treatment

A combination of aerobic (nitrification) and anaerobic (denitrification) treatment may be operated to remove N from slurry. In The Netherlands, this is commonly used for veal calf slurry, due to low acceptability of this type of slurry to arable farmers. Based upon work by Burton et al.[62] and Willers et al.[63], potential $N_2O$ emission from aerobic slurry treatment may be 10 to 20% of the slurry N. The relatively high potential for $N_2O$ emissions during aerobic/anaerobic slurry treatment is related to oxygen

**TABLE 3**
**$N_2O$ Emissions (kg per Animal Place per year) from Pig Housing Systems**

| Fattening pigs housing system | $N_2O$ emission | Reference |
|---|---|---|
| Fully or partly slatted floor | 0.15 | 58 |
| without straw | 0.31 | 59 |
| Deep litter | 2.48 - 3.73 | 57 |
| | 0.59 – 3.44 | 58 |
| | 1.09 | 59 |

limitation of nitrifying bacteria and/or $O_2$ inhibition of denitrifiers[63,64].

Treatment of manure through composting is mainly conducted in poultry husbandry (laying hens), and occasionally on pig and cattle farms (straw-based housing systems), with the aim of obtaining a biologically stable organic fertiliser with a high dry matter and nutrient content. Composting can be operated with either natural or forced aeration. In natural composting systems, air (and thus oxygen) is allowed to enter the compost heaps, whereas during forced composting, air is moved through the stored manure. Hüther et al.[65] conducted experiments with the forced aeration of various types of FYM and reported maximum loss of $N_2O$-N was 1.5% of total N, occurring at aeration rates of around 1.8 $m^3$ of air per h per $m^3$ of FYM, whereas N loss drastically decreased at greater aeration rates (1.8 to 4.8 $m^3$ of air per h per $m^3$ of FYM).

### Options for Control of $N_2O$ Emissions from Animal Housing Systems

With the increased pressure for animal housing systems which take animal welfare into account, straw-based housing systems are currently being developed and introduced in practice (e.g., for pigs[66]). This implies the production of more FYM and consequently an increase in greenhouse gas emissions during storage (with natural composting) and treatment (e.g., forced composting). Improved knowledge of the optimal process conditions during composting, relative to the emissions of (greenhouse) gases, and the development of composting systems operated under optimal conditions, may limit the potential increase in greenhouse gas emissions. Also, for aerobic/anaerobic slurry treatment systems, a better understanding of process conditions leading to advanced process control systems (oxygen supply, technological support) may limit the increased potential for greenhouse gas emissions[64].

## TOWARD SUSTAINABLE LIVESTOCK PRODUCTION

Livestock production in The Netherlands is faced with many regulations. Besides regulations with respect to the emissions to air, soil, and water, other issues are also becoming urgent, e.g., improvements in food safety, healthy food, disease control, animal welfare. Farmers urgently need farm control systems in order to sustain control of the environmental aspects[67].

For this reason, we have started several projects in The Netherlands, where research is being conducted with the goal of sustainable technology development. Therefore, projects must be supported by a broad group of stakeholders. For the pig industry, a multidisciplinary approach means conducting a project with close cooperation between research institutions and private companies. In such an approach, system development moves along the chain from animal feed, via digestion by pigs in innovative housing systems (health, welfare), to fertiliser production from faeces and urine. Sustainability of the system can then be evaluated by a life cycle analysis method based on building materials, mineral management, volatile emissions, and energy and water inputs[68]. Currently, one integrated project in dairy husbandry involves nutrient management.

| | | |
|---|---|---|
| Received: | July | 27, 2001 |
| Revised: | October | 25, 2001 |
| Accepted: | October | 29, 2001 |
| Published: | November | 27, 2001 |
